# Endometriosis and Infertility: A Multi-cytokine Imbalance *Versus* Ovulation, Fertilization and Early Embryo Development

**DOI:** 10.1080/17402520500125484

**Published:** 2005-06

**Authors:** S. Vassiliadis, K. Relakis, A. Papageorgiou, I. Athanassakis

**Affiliations:** 1 Department of Biology University of Crete, Heraklion Crete Greece; 2 Department of Gynecology and Obstetrics University of Crete Hospital, Heraklion Crete Greece

## Abstract

Endometriosis is tightly linked to infertility which is manifested at very early or more advanced stages of the gestational cycle. Alteration on the production of a great number of cytokines/growth factors can be accused for problems on ovum maturation, fertilization or implantation. Yet, macroscopically these stages are characterized by the inability of conception. A closer look of the cytokinic profile during the conceptional and early gestational cycle could, however, localize the problem and allow a therapeutic approach. In this commentary, going through the cytokine requirement during ovulation, fertilization and the early stages of pregnancy, it became possible to specifically define the harmful endometriosis-induced cytokines for each of the conceptional and early gestational stages. Thus, regulating the levels of interferon-γ and tumor necrosis-α will facilitate ovulation and fertilization, whereas adjusting the levels of interleukin-1β and colony stimulating gactor-1 will facilitate implantation.


*Clinical & Developmental Immunology*, June 2005; 12(2): 125–129

Taylor & Francis

Taylor & Francis Group

**MINI-REVIEW**

### Endometriosis and infertility: A multi-cytokine imbalance *versus* ovulation, fertilization and early embryo development

S. VASSILIADIS[1], K. RELAKIS[2], A. PAPAGEORGIOU[2], & I. ATHANASSAKIS[1]

[1]*Department of Biology, University of Crete, Heraklion, Crete, Greece, and* [2]*Department of Gynecology and Obstetrics, University of Crete Hospital, Heraklion, Crete, Greece*

**Abstract**
Endometriosis is tightly linked to infertility which is manifested at very early or more advanced stages of the gestational cycle. Alteration on the production of a great number of cytokines/growth factors can be accused for problems on ovum maturation, fertilization or implantation. Yet, macroscopically these stages are characterized by the inability of conception. A closer look of the cytokinic profile during the conceptional and early gestational cycle could, however, localize the problem and allow a therapeutic approach. In this commentary, going through the cytokine requirement during ovulation, fertilization and the early stages of pregnancy, it became possible to specifically define the harmful endometriosis-induced cytokines for each of the conceptional and early gestational stages. Thus, regulating the levels of interferon-$\gamma$ and tumor necrosis-$\alpha$ will facilitate ovulation and fertilization, whereas adjusting the levels of interleukin-1$\beta$ and colony stimulating gactor-1 will facilitate implantation.

**Keywords:** *Endometriosis, fertilization, cytokine, implantation*

Endometriosis by definition concerns the migration, implantation, growth and function of endometrial tissue mainly to the peritoneum, on or next to the ovaries and less frequently to the cul-de-sac, utero-sacral ligaments, fallopian tubes, vagina, urinary track (20% of cases) or even to the gastrointestinal tract (12–37% of patients) and rarely to the lungs, arms or thighs. The endometrioma once successfully implanted mimics the functions of the physiologic endometrium and follows menstruation. Thus, every month endometriomas fill with blood, thicken, break down and bleed. Yet, in the absence of a natural root for the blood to exit, the implants develop into endometriotic cysts, spots or patches, which may grow and reseed as the menstrual cycle continues. Although endometriomas are not cancerous they can develop and attach to nearby organs. Symptoms of endometriosis, depending on the localization of the implants, include gradually increasing pain to the lower abdomen during or before menstruation, dysparenia, disuria, hypo- or hyper-blood release during menstruation and infertility, which accompanies 75% of the patients. Assuming that the fallopian tubes are permissive and ovulation occurs normally, an important mechanical cause for infertility includes the limited elasticity of organs, which diminishes the possibility of ovum uptake by the fallopian tubes (Lebovic et al. 2001). An additional and perhaps more important parameter to infertility is the cytokine imbalance induced by endometriomas.

### Cytokines in endometriosis

Endometriomas, by mimicking the functions of the physiologic endometrium, are subjected to a similar hormonal regulation ensuring thus thickening and development of blood vessels. Since, however, such changes do not occur in the privileged site of the uterus, they trigger systemic mechanisms to the female organism leading to significant changes of the cytokinic profile. Thus, interleukin-1$\beta$ (IL-1$\beta$) and the vascular endothelial growth factor (VEGF) increase in the serum of endometriotic women possibly to facilitate angiogenesis

Correspondence: I. Athanassakis, Department of Biology, University of Crete, P.O. Box 2208, Heraklion 714-09, Crete, Greece. Tel: 30 2810394355. Fax: 30 2810394379. E-mail: athan@biology.uoc.gr

ISSN 1740-2522 print/ISSN 1740-2530 online © 2005 Taylor & Francis Group Ltd
DOI: 10.1080/17402520500125484

(Wu and Ho 2003). Inteleukin-6 (IL-6) also increases in case of endometriosis ensuring proliferation of endometrial cells, stimulates decidualization but becomes toxic to the late embryo (Wu and Ho 2003). The increased levels of interleukin-8 (IL-8) attract neutrophils to the endometriotic sites and facilitate angiogenesis (Wu and Ho 2003). By the same token, interleukin-10 (IL-10) and colony stimulating factor-1 (CSF-1) increase in the serum of endometriotic women probably due to high numbers of activated macrophages in the endometrium (Wu and Ho 2003, Fukaya et al. 1994). Granulocyte-macrophage colony stimulating factor (GM-CSF) is also found to increase in endometriosis, especially during the secretory phase of the menstrual cycle (Wu and Ho 2003). The increased levels of tumor necrosis factor-α (TNF-α) during endometriosis ensure agglutination of endometrial cells to the epithelium (Wu and Ho 2003). Similarly, interferon-γ (IFN-γ) is also found increased in the serum and peritoneal fluid of endometriotic women (Wu and Ho 2003). Interleukin-15 (IL-15) is known to induce production of IFN-γ and increases in the peritoneal fluid of endometriotic women as well (Arici et al. 2003).

In addition to cytokines, another family of immune molecules found to be disturbed during endometriosis includes soluble HLA molecules. The levels of soluble class-I and class-II HLA proteins are found to significantly decrease in the serum of women suffering from endometriosis (Matalliotakis et al. 2001). Yet, there is no information on whether and how these molecules can affect the early gestational stages.

### Cytokines in ovulation, fertilization and early embryo development

Cytokines are still believed to be very important regulatory molecules not only for the immune system, but for many other significant functions of the organism. Yet, the complicated mechanisms of their regulation, including regulation of their cellular receptors, their synergistic and/or antagonistic interactive nature and their differential production upon the various cellular stimuli, make these molecules very difficult to study and more importantly very complicated to use in therapeutic treatments.

The reproductive process is now known to be directly linked to the cytokinic regulation. Thus, during ovulation, gonadotropins (follicular stimulating hormone-FSH- and luteinizing hormone-LH-) simultaneously activate production of the plasminogen activator with both TNF-α and IL-1β and lead to follicular rupture (Figure 1). The plasminogen activator pathway goes through activation of plasmin. In turn, plasmin stimulates collagenases, which actually induce rupture of the follicle and ovulation.

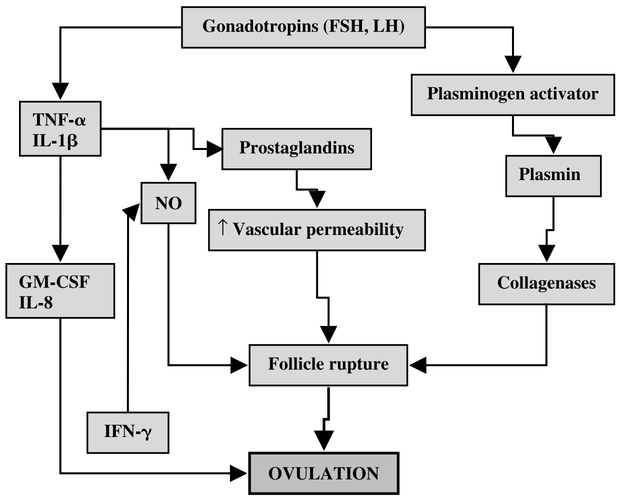

Figure 1. Hormones and cytokines involved in the process of ovulation.

On the other hand, TNF-α and IL-1β are involved in two different pathways leading to the follicular rupture (Machelon and Emilie 1997, Adashi 1990, Adashi 1998). The first is via prostaglandins which, by increasing vascular permeability, facilitate the follicular rupture and the second is via nitrogen oxide (NO) leading directly to follicular rupture. By the same token, TNF-α and IL-1β stimulate production of GM-CSF and IL-8, cytokines that will further facilitate ovulation and embryo implantation (Arici et al. 1996). At that stage, IL-6 and IFN-γ are also being detected in the endometrium possibly accounting for NO production (Athanassakis et al. 1999).

Specifically for IFN-γ, it seems that this agent acts in very narrow timing windows, since it becomes undetectable after the follicular rupture and if present, under not physiological conditions, inhibits estrogen disallowing thus ovulation. At the same time IFN-γ inhibits sperm mobility acting as a negative parameter to fertilization (Machelon and Emilie 1997). Similar effects apply to TNF-α, which although facilitating follicular rupture itself through NO and prostaglandin synthesis, inhibits the plasminogen activity (Karakji and Tsang 1995) and, if present in a later stage of the reproductive cycle, will inhibit steroid biosynthesis, sperm mobility and sperm/mucus interaction (Machelon and Emilie 1997, Sato et al. 1995).

Upon fertilization, although the early embryo produces its own cytokines to support growth, it is tightly dependent on maternal endometrium for implantation (Table I). Thus, the two-cell stage embryo produces small quantities of TNF-α and IFN-γ possibly to initiate an inflammatory reaction to the endometrium preparing implantation. However, high concentrations of IFN-γ and TNF-α are toxic to the two-cell stage embryos. At the stage of blastocyst the trophectoderm produces IL-10, IL-3 and IL-6, the zona pellucida produces GM-CSF

Table I. Cytokine production at different stages of early embryo development.

| Stage of embryo development | Cytokines |
|---|---|
|  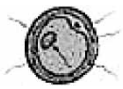 Fertilisation | • TNF-α inhibits sperm mobility and sperm/mucus interaction<br>• IFN-γ inhibits sperm mobility |
|  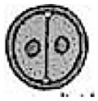 Two-cell embryo | • Produces small quantities of TNF-α and IFN-γ<br>• At higher concentrations TNF-α and IFN-γ become toxic |
|   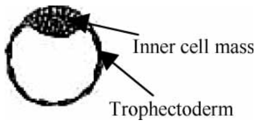 Blastocyst | • Trophectoderm: IL-3, IL-6, IL-10<br>• Inner cell mass: IL-10<br>• Zona pellucida: GM-CSF |
|  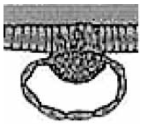 Embryo implantation | • Endometrium: GM-CSF, IL-3, IL-10, IL-6<br>• Mesometrium: IL-1, CSF-1, TNF-α, IFN-γ<br>• Myometrium: IL-10, IL-6 |

and the inner cell mass also produces IL-10 (Athanassakis et al. 1999). Among these cytokines, IL-10, IL-3 and GM-CSF facilitate embryo growth (Athanassakis et al. 1987) whereas IL-6 is involved in the inflammatory reaction during implantation. During the preparative stage of embryo implantation, GM-CSF and IL-3 are present in the endometrium, IL-10 in the endometrium/ myometrium, IL-1 and CSF-1 are produced by the mesometrium, uterine epithelium and endometrium, TNF-α and IFN-γ are present in the mesometrium and IL-6 is found to be also produced by endometrium and is present in the endometrium/myometrium during implantation (Adashi 1998, Araki et al. 1996).

Once implantation is completed, TNF-α and IFN-γ will play an important role in trophoblast cell differentiation/maturation (Athanassakis et al. 2000), whereas IL-6 will induce decidualization in the uterus (Machelon and Emilie 1997). All these cytokines are harmful to the late embryo and at higher concentrations induce fetal abortion. On the other hand, GM-CSF, CSF-1 and IL-3 improve trophoblast proliferation and function (Athanassakis et al. 1987), whereas IL-10 promotes late embryo development (Wegmann et al. 1993).

### Endometriosis and cytokine-induced infertility

Considering the cytokine pathology during endometriosis and the cytokine requirement for ovulation, fertilization and early embryo development, it is not difficult to foresee how endometriosis correlates to infertility (Table II). However, if one screens all cytokines induced in endometriosis one by one, it will be realized that only four to up-to-now knowledge cytokines are mainly responsible for disallowing conception. Namely, the increased levels of IFN-γ and TNF-α in the serum of endometriotic women, although facilitating follicular rupture, inhibit ovulation by acting as negative regulators on steroid biosynthesis [especially estrogen; Machelon and Emilie 1997, Karakji and Tsang 1995, Terranova and Rice 1997]. Although not fully explained, there are reports in the literature showing that the ovarian function is altered in significant number of fertile women with endometriosis (Winglfield et al. 1994, Bulletti et al. 2002). These two factors will also exert a negative effect on fertilization since they will decrease sperm mobility while TNF-α will also inhibit sperm/mucus interaction. Even if the ovum succeeds to get fertilized, the elevated concentrations of these two cytokines will be toxic to the two-cell stage embryo. IL-8 which, as previously described, exerts a positive effect in ovulation, in most cases of women can not apparently overcome the negative effects of the elevated IFN-γ and TNF-α concentration.

The next two cytokines, that negatively affect these early stages of pregnancy establishment, are IL-1β and CSF-1 acting as embryo implantation inhibitors (Araki et al. 1996, Terranova and Rice 1997). As discussed earlier, IL-1β is inducing ovulation by facilitating follicular rupture but, if its production in the uterus persists, it inhibits embryo implantation. Similarly, CSF-1 facilitates ovulation and promotes trophoblast growth of the late embryo but, always within the narrow timing of implantation, it exerts

Table II. Comparative effect of cytokines in endometriosis and pregnancy.

| | Endometriosis | Pregnancy |
|---|---|---|
| IL-1β | • Increases<br>• Promotes angiogenesis | • Necessary to ovulation<br>• Implantation inhibitor |
| IL-2 | • Increases | • Stable levels |
| IL-3 | Information not available | • Promotes implantation<br>• Promotes growth/function of mature trophoblasts |
| IL-6 | • Increases<br>• Promotes growth of endometrial cells | • Activates decidualization<br>• Harmful to late embryo |
| IL-8 | • Increases<br>• Attracts neutrophils<br>• Promotes angiogenesis | • Necessary to ovulation |
| IL-10 | • Increases | • Promotes fetal development |
| CSF-1 | • Increases | • Necessary to ovulation<br>• Implantation inhibitor<br>• Promotes growth/ function of mature trophoblasts |
| GM-CSF | • Increases in endometrial cells especially during the secretory phase | • Necessary to ovulation<br>• Promotes implantation<br>• Promotes growth/function of mature trophoblasts |
| TNF-α | • Increases<br>• Facilitates adherence of endometriotic cells to peritoneal mesothelium | • Inhibits steroid biosynthesis<br>• Inhibits sperm mobility<br>• Inhibits sperm/mucus interaction<br>• Toxic to early (2-cell) and mature embryo (12–20 weeks) |
| IFN-γ | • Increases | • Inhibits sperm mobility<br>• Promotes trophoblast differentiation<br>• Toxic to early (2-cell) and mature (12–20 weeks) embryo |

negative feedback mechanisms disallowing embryo anchorage to the endometrium.

If, however, implantation occurs, all the other cytokines known to be induced up-to-now in endometriotic women have positive effects on embryo development. Namely, GM-CSF, CSF-1 and IL-10 will stimulate trophoblast growth/ function and embryo development (Athanassakis et al. 1987, Athanassakis-Vassiliadis et al. 1993).

Indeed, this inability to conception and early embryo loss in endometriotic women explains the lack of correlation between endometriosis and recurrent abortion.

Recurrent abortions have been correlated to cytokine imbalance as well as to disturbance of soluble HLA levels in the maternal serum (Athanassakis et al. 1999) and are defined as the occurrence of three or more clinically recognized fetal losses before the 20th week of pregnancy when no gynecological, pathological or genetic problems have been detected. Based on the definitions given above, endometriosis and recurrent abortions do not seem to have a direct correlation. Various studies have tried to examine this issue, yet because of the lack of valid controls, it has become difficult to evaluate the described results. In contrast to only one study, most reports fail generally to establish a correlation between endometriosis and recurrent abortion (Vercammen and D'Hooghe 2000). Thus, Vercammen and D'Hooghe 2000, Marcoux et al. 1997 have shown that fetal losses occur with the same frequency in endometriotic women with or without surgical treatment of endometriosis. Similarly, Matorras and Rodriguez 1998 showed that 7% of their patients with endometriosis underwent recurrent abortion as compared to 6% of recurrent abortions in patients without endometriosis. In other groups of patients, 45% of women with endometriosis were also suffering from recurrent abortions, whereas 34% of their recurrent aborters did not have any signs of endometriosis (Vercammen and D'Hooghe, 2000). In general, many reports claim that the occurrence of recurrent abortions does not show to increase in women suffering from endometriosis, probably due to the fact that infertility in endometriotic women occurs during the early conceptional and gestational stages (FitzSimmons et al. 1987, Pittaway et al. 1988). However, if an endometriotic woman successfully overcomes the early gestational stages she could develop symptoms of recurrent abortion because of the imbalance of cytokines and soluble HLA antigens already established to her organism.

### Conclusion

As it can be realized, the cytokine induced infertility in endometriotic women is a multi-parameter event. Not all women develop the same levels of cytokines at the same time. Ovulation, fertilization and embryo development may occur in the short-timed windows as far as cytokine requirements are fulfilled. These events, however, become a difficult task to achieve in endometriotic women. Depending on the stage of endometriosis severity, the site of endometrioma implantation and the general pathology of the organism, the ideal approach would be to specifically modulate the cytokine profile in order to allow ovulation, fertilization and implantation to occur. Although this issue has not yet been experimentally investigated, it is likely that following the cytokinic profile during the menstrual cycle of endometriotic women will determine the cytokines contributing to the negative feedback mechanisms. Such diagnosis could lead to a specific intervention and thus treatment of the woman by using pharmaceutically approved cytokine inhibitors, antagonists, humanized antibodies, etc. In particular, it seems that the initial regulation of IFN-$\gamma$ and TNF-$\alpha$ followed by the regulation of IL-1$\beta$ and CSF-1 is the most important issue to deal with, since as mentioned above, these two are the up-to-now known cytokines that interfere with the first events of the conceptional and gestational cycle.

### References

Adashi EY. 1990. The potential relevance of cytokines to ovarian physiology; the emerging role of resident ovarian cells of the white blood series. Endocr Rev 11:454–464.

Adashi EY. 1998. The potential role of interleukin-1 in the ovulatory process: A evolving hypothesis. Mol Cell Endocrinol 140:77–81.

Araki M, Fukumatsu Y, Katabuchi H, Shultz LD, Takahashi K, Okamura H. 1996. Follicular development and ovulation in macrophage colony-stimulating factor-dependent mice homozygous for the osteopetrosis (*op*) mutation. Biol Reprod 54:478–484.

Arici A, Oral E, Bukulmez O, Buradagunta S, Engin O, Olive DL. 1996. Interleukin-8 expression and modulation in human preovulatory follicles and ovarian cells. Endocrinology 137:3762–3769.

Arici A, Matalliotakis I, Goumenou A, Koumantakis G, Vassiliadis S, Selam B, et al. 2003. Increased levels of interleukin-15 in the peritoneal fluid of women with endometriosis: Inverse correlation with stage and depth of invasion. Human Reprod 18:429–432.

Athanassakis I, Bleackley RC, Paetkau V, Guilbert L, Barr PJ, Wegmann TG. 1987. The immunostimulatory effect of T cells and T cell lymphokines on murine fetally derived placental cells. J Immunol 138:37–44.

Athanassakis I, Farmakiotis V, Papadimitriou L. 1999. Uterine cytokine production during the menstrual cycle and preimplantation stages in mice. Dev Immunol 7:33–42.

Athanassakis I, Paflis M, Ranella A, Vassiliadis S. 1999. Detection of soluble HLA-G levels in maternal serum can be predictive for a successful pregnancy. Transplant Proc 31:1834–1837.

Athanassakis I, Papadimitriou L, Bouris G, Vassiliadis S. 2000. IFN-gamma induces trophoblast differentiation of ectoplacental cone cells. Dev Comp Immunol 24:663–672.

Athanassakis-Vassiliadis I, Papamatheakis J, Vassiliadis S. 1993. Specific CSF-1 binding on murine placental trophoblasts and macrophages serves as a link to placental growth. J Recept Res 13:739–751.

Bulletti C, De Ziegler D, Polli V, Del Ferro E, Palini S, Flamigni C. 2002. Characteristics of uterine contractility during menses in women with mild to moderate endometriosis. Fertil Steril 77:1156–1161.

FitzSimmons J, Stahl R, Gocial B, Shapiro SS. 1987. Spontaneous abortion and endometriosis. Fertil Steril 47:696–698.

Fukaya T, Sugawara J, Yoshida H, Yajima A. 1994. The role of macrophage colony stimulating factor in the peritoneal fluid in infertile patients with endometriosis. J Exp Med 172:221–226.

Karakji EG, Tsang BK. 1995. Tumor necrosis factor alpha inhibits rat granulosa cell plasminogen activity *in vitro* during follicular development. Biol Reprod 52:745–752.

Lebovic DL, Mueller MD, Taylor RN. 2001. Immunobiology of endometriosis. Fertil Steril 75:1–10.

Machelon V, Emilie D. 1997. Production of ovarian cytokines and their role in ovulation in the mammalian ovary. Eur Cyt Netw 8:137–143.

Marcoux S, Maheux R, Berube S. 1997. and the Canadian Collaborative Group on Endometriosis. Laparoscopic surgery in infertile women with minimal or mild endometriosis. N Engl J Med 337:217–222.

Matalliotakis I, Athanassakis I, Neonaki M, Goumenou A, Vassiliadis S, Koumantakis E. 2001. The possible anti-inflammatory role of circulating human leukocyte antigen levels in women with endometriosis after treatment with danazol and leuprorelin acetate depot. Med Inflamm 10:75–80.

Matorras R, Rodriguez F, Gutierrez de Teran G, Pijoan JI, Ramon O, Rodriguez-Escudero FJ. 1998. Endometriosis and spontaneous abortion rate: A cohort study in infertile women. Eur J Obstet Gynecol Reprod Biol 77:101–105.

Pittaway DE, Vernon C, Feyez JA. 1988. Spontaneous abortions in women with endometriosis. Fertil Steril 50:711–715.

Sato E, Nakayama T, Kamio K, Takahashi Y, Toyoda Y. 1995. Immunohistochemical localization and possible roles of tumor necrosis factor-alpha in mouse cumulus-oocyte complexes. Dev Growth Differ 37:413–420.

Terranova PF, Rice VM. 1997. Review: Cytokine involvement in ovarian processes. Am J Reprod Immunol 37:50–63.

Vercammen EE, D'Hooghe TM. 2000. Endometriosis and recurrent pregnancy loss. Semin Reprod Med 18:363–368.

Wegmann TG, Lin H, Guilbert L, Mosmann TR. 1993. Bidirectional cytokine interactions in the materno-fetal relationship: Successful allopregnancy is a Th2 phenomenon. Immunol Today 14:353–355.

Winglfield M, O'Herlihy C, Finn MM, Tallon DF, Fottrell PF. 1994. Follicular and luteal phase salivary progesterone profiles in women with endometriosis and infertility. Gynecol Endocrinol 8:21–25.

Wu M-Y, Ho H-N. 2003. The role of cytokines in endometriosis. Am J Reprod Immunol 49:285–296.

